# Help-Seeking Undocumented Migrants in the Netherlands: Mental Health, Adverse Life Events, and Living Conditions

**DOI:** 10.1007/s11013-022-09790-5

**Published:** 2022-07-30

**Authors:** Sandrine J. C. Vollebregt, Willem F. Scholte, Annette Hoogerbrugge, Koen Bolhuis, Jentien M. Vermeulen

**Affiliations:** 1 Doctors of the World, Amsterdam, The Netherlands; 2 Amsterdam UMC, Department of Psychiatry, University of Amsterdam, Amsterdam, The Netherlands; 3 Department of Child and Adolescent Psychiatry/Psychology, Erasmus Medical Centre, Rotterdam, The Netherlands; 4 Utrecht, The Netherlands

**Keywords:** Undocumented migrants, Mental health, Psychological distress, Trauma, Life events

## Abstract

Undocumented migrants are a particularly vulnerable group regarding (mental) health, living conditions, and restricted access to health care. The aim and objective of the study was to examine the prevalence and correlates of mental health problems in a help-seeking population of undocumented migrants. Observational study was performed by integrating cross-sectional questionnaire data with retrospective electronic patient record data. Undocumented migrants attending medical and psychological consultation hours of a Netherlands-based non-governmental organization completed the Self-Reporting Questionnaire (SRQ). We examined scores of the instrument’s 24 items version (SRQ-24) and its 20 items version (SRQ-20). Correlates of mental health were estimated using parametric tests. On the SRQ-20, 85% (95% CI 77–91%) of the sample (*N* = 101) scored ≥ 8, the clinical cut-off value for common mental disorders; mean = 12.4 ± 4.6, range 0–20. Adverse life events like physical and sexual assault were reported in 37% of the medical records (*N* = 99) and had a medium-to-large effect (Cohen’s *d* = 0.76) on SRQ-24 scores. Mental health problems are common in help-seeking undocumented migrants. This study underlines the need of improving access to mental health care for undocumented migrants.

## Introduction

Approximately 23,000–58,000 undocumented migrants (UMs) reside in the Netherlands according to the latest estimate of 2017–2018 (Heijden et al., 2020). Most of the UM population, about 10,000–30,000 people, live in Amsterdam, the capital city of the Netherlands (Municipality of Amsterdam 2021). Lives of UMs are characterized by unstable living conditions, fear of being arrested, low levels of emotional support, and poor (mental) health (Chauvin and Simonnot 2013; Lahuis et al. 2019; Rietveld et al. 2019; Woodward et al. 2014). However, quantitative data on mental health of UMs are scarce and often biased by selection and attrition, as UMs are notoriously difficult to include in regular clinical research. Most research performed on UMs is therefore qualitative in nature. According to the ‘inverse care law’, “the availability of good medical care tends to vary inversely with the need for it in the population served” (Hart 1971). It is to be expected that such a law will apply to mental health care for UMs, which makes it vital to recollect further data on mental care in order to assess their needs. This study contributes to this and sheds light on an otherwise poorly visible population by targeting UMs visiting a low-threshold health care program of Doctors of the World (DotW), an NGO that aids UMs with acces to health care.

## Living Conditions and (Mental) Health

UMs constitute a heterogenous group, comprised of rejected asylum seekers, individuals who have not (yet) submitted an application for asylum, and overstayers of visas or work permits (HUMA Network 2009; Heijden et al. 2020). As it has been shown by a multi-country survey study carried out through Europe (Chauvin and Simonnot 2013), the legal position of UMs leads to a precarious existence. As living and working legally is highly inaccessible for UMs in many countries, their housing and working conditions remain unstable. Almost half of the population has no income from work (Chauvin and Simonnot 2013), and when UMs are able to get access to work, it is usually through exploitation on the black market (Myhrvold et al. 2017). Furthermore, UMs are often isolated from the host population, and experience low levels of emotional support (Chauvin and Simonnot 2013). These social inequalities are associated with an increased risk of common mental disorders (World Health Organization and Calouste Gulbenkian Foundation 2014).

There is ample evidence on the prevalence of potentially traumatic experiences in UMs as a group. A study among rejected Iraqi asylum seekers in Denmark showed an average of 8.5 traumatic events before arrival in the host country (Schwarz-Nielsen and Elklit 2009). A large systematic review assessing forced migrants yielded prevalence rates of torture and other war-related traumatic events ranging from 1 to 76% (median 27%) (Sigvardsdotter et al. 2016). It has been shown that such events often have negative mental health consequences, e.g. post-traumatic stress disorder, depressions, and anxiety disorders (Fazel et al. 2005; Steel et al. 2009). Such outcomes may similarly apply to UMs, a substantial proportion of whom have had long and difficult journeys to the host country during which they might have faced violence (Chauvin and Simonnot 2013; Fargues 2017), or dangerous sea crossings (Fargues 2017). Similarly, it is expected that a great number of UMs might have escaped violence and repression (Chauvin and Simonnot 2013), and that they will be vulnerable to sexual and gender-based violence in Europe (Keygnaert et al. 2015).

A scoping review shows that UMs report poor health, being mental health problems particularly common (Woodward et al. 2014). According to UMs, their mental health problems are experienced as a direct result of their poor living conditions and undocumented status (Cha et al. 2019; Teunissen et al. 2014a, b). Similarly, Dutch health care professionals confirm that stress, traumas, sleeping problems, and gloominess are more common in UMs than in the general population (Veenema et al. 2012; Rietveld et al. 2019).

## Access to (Mental) Health Care

The right to health, meaning ‘the right of everyone to enjoy the highest attainable standard of physical and mental health’, is outlined in several treaties in the human rights framework. However, across EU member states, policies regulating UMs’ access to health care differ widely. These policies range from less than minimum rights, meaning having to pay for emergency care and no access to primary and secondary care (e.g. Sweden and Bulgaria) to rights similar to documented citizens, meaning access to primary, secondary, and preventative health care free of charge (e.g. Italy, France, and the Netherlands) (Cuadra 2012).

In the Netherlands, Dutch professional guidelines state that medical doctors should provide ‘responsible and appropriate care’, and that doctors should follow the same medical guidelines for UMs as for documented Dutch citizens. The health care that UMs are entitled to corresponds almost completely to what is covered by Dutch basic health insurance. This includes primary health care, specialized health care, most medication, pregnancy care, and mental health care. In vitro fertilization, sex reassignment treatment, and certain dental care are excluded from this entitlement (Commissie Medische zorg voor (dreigend) uitgeprocedeerde asielzoekers en illegale vreemdelingen 2007).

Compared to some other countries, the legal rights for UMs in the Netherlands regarding health care are relatively good. But formal rights are of little value unless they can be realized. In practice, access to Dutch health care is greatly impaired for UMs (College voor de Rechten van de Mens et al. 2015; Hintjens et al. 2020; Teunissen et al. 2015; Veenema et al. 2012). This access problem can be attributed to policy, the health system, and individual barriers (Hacker et al. 2015).

## Policy Barriers

Paradoxically, even if UMs are entitled to (almost all) care that is covered by basic insurance, they are excluded from having a Dutch health insurance. This is determined by the Linkage Act, a law implemented in 1998 to exclude UMs from public services (HUMA Network 2009). As a consequence, UMs must in principle pay for health care costs themselves. However, when a UM states that he or she is unable to pay (which is often the case), health care providers can get 80% of their costs reimbursed through a governmental fund. For pregnancy and childbirth care, a 100% financial reimbursement is possible (Biswas et al. 2012; CAK 2021). This procedure, nonetheless, leads to complexity and extra paper work, which is a proven barrier for health care providers to deliver care as usual. (Goossens and Depoorter 2011; Hacker et al. 2015; Moons 2021).

## Health System Barriers

Dutch health care can be characterized by the central role of the General Practitioner (GP) who offers comprehensive primary health care, including basic mental health care. GP care is 24/7 available, and it constitutes the entry point to secondary (specialized) health care. Secondary care is only accessible through referral by a GP, unless it concerns a life-threatening emergency. Being registered with a GP is thus very important, and everyone working and living in the Netherlands is supposed to be registered with a GP, who keeps track of one’s complete medical history (including both physical and mental health). Theoretically, UMs are also able to register with a GP, but in practice many lack registration. In a recent study performed by DotW among GPs in Amsterdam, 41.2% of surveyed GPs stated to sometimes refuse to register or treat UMs (Moons 2021). Also in secondary care, it happens that UMs are denied care by hospital staff, as they wrongly assume that UMs do not have a right to health care if they cannot pay (Hintjens et al. 2020). Other barriers that UMs encounter are forced cash payment or invoices being sent, which prevents them from seeking care in the future (Schoevers et al. 2010). UMs also report experiencing neglect and indifference from health care providers, or even active hostility (Hintjens et al. 2020). Other potential reasons contributing to UMs not receiving proper care include a lack of cultural sensitivity of health care providers and language barriers (Hintjens et al. 2020). The costs of professional interpreters are not reimbursed by the government, except for interpretation services in mental health care for adults.

## Individual Barriers

In the Netherlands, there is a tendency of UMs to avoid seeking health care, unless it is an emergency (De Nationale Ombudsman 2013). On an individual level, one of the most important barriers is the widespread fear of UMs being reported to the police or the immigration authorities (Goossens and Depoorter 2011; Hacker et al. 2015; Hintjens et al. 2020; Teunissen et al. 2014a, b). Another reason for UMs to not seek appropriate health care is their lack of knowledge of their right to health care and of the health care system (Dorn et al. 2011; Hintjens et al. 2020; Schoevers et al. 2010). In addition, poor language proficiency of the language of the host country imposes difficulties in expressing one’s health care needs. Finally, their precarious financial and living conditions complicate access to health care. The lack of financial resources makes it difficult to pay for transport in order to reach medical services (Goossens and Depoorter 2011; Hintjens et al. 2020). Furthermore, due to the fact that most UMs do undeclared labour, taking time off to visit a doctor becomes an almost impossible endeavour (Hintjens et al. 2020).

## Mental Health Care

Specific barriers to mental health care comprise the insufficient knowledge of UMs about the role of GP’s, and lack of trust in GPs’ competencies regarding mental health prevent them from visiting a GP, while the GP forms the gateway to mental health care (Teunissen et al. 2014a, b). Furthermore, shame and stigma surrounding mental health problems create a hesitancy to seek help (Teunissen et al. 2014a, b; Veenema et al. 2012). In addition, UMs often consider mental health care unlikely to be beneficial in their situation, as they relate their mental health problems directly to their legal status. Mental health care is seen as a last resort, after seeking support from friends or in religion (Cha et al. 2019; Teunissen et al. 2014a, b). GPs report to perceive frequent somatic presentations of mental health problems and the high number of other physical and social problems as barriers to deliver appropriate care (Teunissen et al. 2015). Taken together, these factors lead to a strongly impaired access to mental health care for UMs in the Netherlands, even while evidence points towards a high prevalence of mental health problems in this population (Priebe et al. 2016; Woodward et al. 2014).

## Aims and Objectives

This research was initially set up in the context of determining whether there was a need for an additional service of DotW: a Psychosocial Support Program. The aims of this study were (1) to examine the prevalence of mental health problems in UMs in a help-seeking setting in the Netherlands, (2) to identify possible correlates of mental health, and (3) to indicate whether there was a need for psychosocial support. It was hypothesized that mental health problems were common and related to poorer living conditions and adverse life events. Since preliminary results showed a high burden of psychological complaints, the Psychosocial Support Program was set up in June 2017.

## Methods

### Setting: Doctors of the World

**Fig. 1:**
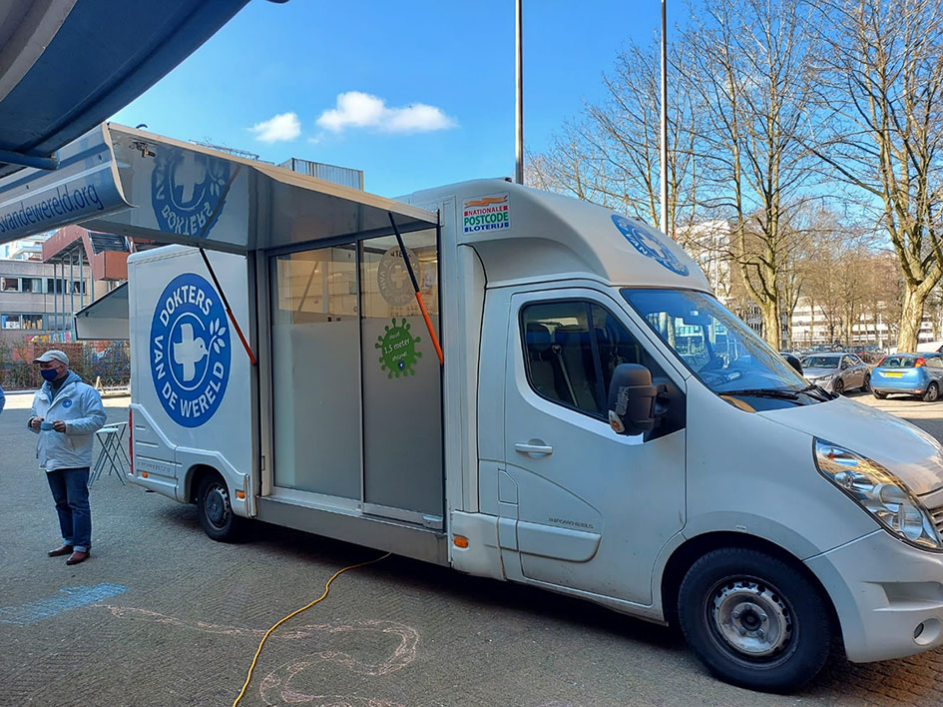
The mobile converted minivan where consultations of the general medical consultation hour take place. The minivan visits places in Amsterdam and Rotterdam (two major cities in the Netherlands) where UMs reside, like night shelters, squats, a community centre for UMs, and central places in certain neighbourhoods. When volunteers visit these locations, UMs are actively invited to visit the van if they are in need of medical help

NGOs like Doctors of the World (DotW) aim to aid UMs and health care providers to overcome barriers to health care. DotW does so by providing information to UMs and health care providers about the right to health care and the governmental financial reimbursement system. In addition, they organize different kinds of consultation hours specifically for UMs on several locations, thus forming a ‘safety net’. These include the following:

1. *General medical consultation hours.* In these walk-in consultation hours, medical advice is given by rotating volunteering medical doctors. Over-the-counter medication and hygiene products are distributed free of charge. Referrals to GPs, a dentist or emergency services are done when needed. These consultation hours are held in a converted minivan (Fig. 1), which visits places in Amsterdam and Rotterdam (two major cities in the Netherlands) where UMs reside, like night shelters, squats, a community centre for UMs, and central places in certain neighbourhoods. When volunteers visit these locations, UMs are actively invited to visit the minivan if they are in need of medical help.
2. *A Sexual and Reproductive Health consultation hour.* In these walk-in consultation hours, advice is given on gynaecological issues, and diagnostics and treatment of STDs are available. Referrals to GPs or midwives are given when needed.
3. *A Psychosocial Support Program (PSP).* In this program, volunteering psychologists, medical doctors, and social workers offer basic psychological support and, if needed, refer to specialized mental health services. The PSP consultations are held in the office of DotW in Amsterdam and the office of another support organization in Rotterdam. Usually, people visiting the PSP were referred from the general medical consultation hour.

Respondents of this study were recruited at general medical consultation hours and, later, at the Psychosocial Support Program.

### Participants

A convenience sampling method was used. Recruitment and data collection were performed by the volunteering medical doctors and psychologists. The participants can be divided into two groups:

- *General health care seeking group (n = 47)*: This group consists of UMs included at the general medical consultation hours, before the Psychosocial Support Program came into existence. They were included regardless of their reason of visit (physical and/or psychological), resulting in a total of a sample of 47 respondents who were seeking medical attention.
- *Psychological help-seeking group (n = 54)*: This group consists of UMs included after the start of the Psychosocial Support Program. They were included at the general medical consultation hours because of a psychological problem (n = 16) and at the Psychosocial Support Program (n = 38), resulting in a total of 54 respondents who presented themselves with psychological problems.

### Data Collection

After verbal informed consent was obtained, the Self-Reporting Questionnaire-24 (SRQ-24) was administered. Questions were read aloud in English, Dutch, or French. When needed, additional explanation was given. Patients were included from June 2016 to September 2018. The Psychosocial Support Program was initiated in June 2017. The first author (SV) collected the survey data and extracted data from the medical records.

### Measures

Information on sex, age, country of origin, and living conditions was extracted from the medical records. Furthermore, the records were screened for mental health referrals, psychiatric history (defined as a previously diagnosed psychiatric disorder, current or previous use of psychotropics, a previous psychiatric treatment or a suicide attempt), chronic disease (defined as cardiovascular disease, diabetes, or a malignancy), and substance abuse (alcohol or drugs). In addition, all electronic patient records were screened for the mentioning of adverse life events.

### Self-Reporting Questionnaire (SRQ)

Mental health problems were measured using the Self-Reporting Questionnaire (SRQ), a simple and short screening instrument for common mental disorders (CMD) developed by the World Health Organization for the low- and middle-income primary health care setting (Beusenberg and Orley 1994). It has been used and validated in different populations, including refugee populations (Bojorquez et al. 2015; Martyns-Yellowe 1995; Rahman and Hafeez 2003; Ventevogel et al. 2007).

The SRQ consisted of 24 items. The first twenty items are on neurotic symptoms, which reflect depressive symptoms, anxiety, and psychosomatic complaints. These, taken together as a group, are often referred to as ‘common mental disorders’. The last four items reflect psychotic symptoms. In many studies, only the first 20 items are used (referred to as SRQ-20), in other studies, all 24 items are used (referred to as SRQ-24). For this study, measurement of psychotic symptoms was considered relevant as these may prevail as trauma-related features and, whether present in that quality or as a chronic issue, may be highly debilitating. To facilitate comparison to other published studies, both SRQ-20 and SRQ-24 scores are presented.

SRQ items are scored 0 (‘no’, symptom absent) or 1 (‘yes’, symptom present). A sum score is created by the total number of ‘yes’ answers. Higher scores indicate a higher likelihood of CMD (Beusenberg and Orley 1994). In most studies, a cut-off value of 7–8 on the neurotic items was used for the presence of CMD (Beusenberg and Orley 1994), although validation studies have led to contrasting cut-off values in different population, ranging from ≥ 3 in Ethiopia to ≥ 17 for women in Afghanistan. (Hanlon et al. 2008; Ventevogel et al. 2007). We applied the most used cut-off value of ≥ 8 and a more stringent value of ≥ 13; the latter being similar to the cut-off value used in a study among Afghan mothers caring for children in a refugee camp in Pakistan (Beusenberg and Orley 1994; Rahman and Hafeez 2003).

## Life Events Checklist for DSM-5 (LEC-5)

The Life Events Checklist for DSM-5 (LEC-5) is a self-report measure designed to assess exposure to sixteen events known to potentially result in PTSD or distress (Weathers et al. 2013). This checklist was used to determine which events found in the patient records could be labelled as potentially traumatic, and to categorize these events.

### Analysis

Firstly, descriptive statistics were used to describe the population. Distribution assessment was performed for the SRQ-20 and the SRQ-24 by making a histogram, comparing means with medians, and performing Kolmogorov–Smirnov tests. As scores were distributed normally, means and standard deviations were calculated. The unpaired two-sample t-test was used to compare SRQ scores of the general health care seeking group to those of the psychological help-seeking group. Secondly, Cronbach’s alpha coefficient was calculated to assess internal consistency of the questionnaire. Thirdly, to assess the association between SRQ-20 scores and separate variables, different parametric tests were used: Pearson’s rho (age and length of stay in the Netherlands), one-way ANOVA (living situation and region of origin), and the unpaired two-sample t-test (chronic disease, psychiatric history, substance use, referral to mental health services, and adverse life events in the electronic patient record). Effect sizes were calculated using a Cohen’s d calculator (University of Colorado Colorado Springs 2000). IBM SPSS Statistics version 25 was used for all other statistical analyses (IBM Corp 2017).

### Ethical Procedures

This study has been submitted to the Medical Research Ethics Committee of UMC Utrecht, which granted exemption of the obligation to request official ethical approval, meaning the Medical Research Involving Human Subjects Act (WHO, 2014) did not apply to this study. All procedures performed in this study were in accordance with the ethical standards of the 1964 Helsinki declaration and its later amendments or comparable ethical standards. Respondents were informed about the use of anonymized data for analysis and reporting.

### Results

The convenience sample consisted of 76 males and 25 females, being the median age 36 years (interquartile range = 29–48). Most respondents originated from the Middle East and North Africa (33%), and Eastern Africa (29%). Thirty-five different home countries were reported, of which Somalia (11%), Eritrea (11%), Sudan (8%), and Nigeria (8%) were most common. Most people lived in night shelters (33%) or were lodging with acquaintances, friends, or family (21%). Sixteen percent lived in a squat or another unofficial location and 9% lived on the streets. The median duration of residence in the Netherlands was 7.5 years (interquartile range = 2.3–19.8), but this was only reported in 24% of the cases (Table 1).

**Table 1:** Sociodemographic characteristics

Characteristic	%*	n
Gender		
Male	75.2	76
Female	24.7	25
Age (years)		
18–20	1.0	1
21–30	27.7	28
31–40	32.7	33
41–50	23.8	24
51–60	10.9	11
> 61	4.0	4
Region of origin		
Middle East and North Africa	32.7	33
Eastern Arica	28.7	29
Western Africa	18.8	19
East Asia and Pacific	5.0	5
Europe and Central Asia	5.0	5
Latin America and Caribbean	5.0	5
Central Africa	4.0	4
South Asia	1.0	1
Country of origin		
Somalia	10.9	11
Eritrea	10.9	11
Sudan	7.9	8
Nigeria	7.9	8
Morocco	5.9	6
Algeria	5.9	5
Suriname	4.0	4
Ethiopia	4.0	4
Afghanistan	4.0	4
26 other countries (≤ 3 respondents per country)	40.0	40
Housing conditions		
Night shelter (BBB)**	32.7	33
Lodging with adult acquaintances/friends/family	20.8	21
Squat	15.8	16
On the streets	8.9	9
Social–cultural project***	7.9	8
Unknown	13.9	14

*Due to rounding, the percentages do not always add up to 100%

**Night shelter (Dutch: bed-bad-broodopvang or BBB): a shelter for UMs whose asylum application was rejected, provided by the municipality. It offers basic needs as a dorm, sanitary facilities, and dinner and breakfast

***A former office building rented by small companies, facilitating free living space for 40–50 UMs

### Mental Health Problems

**Fig. 2:**
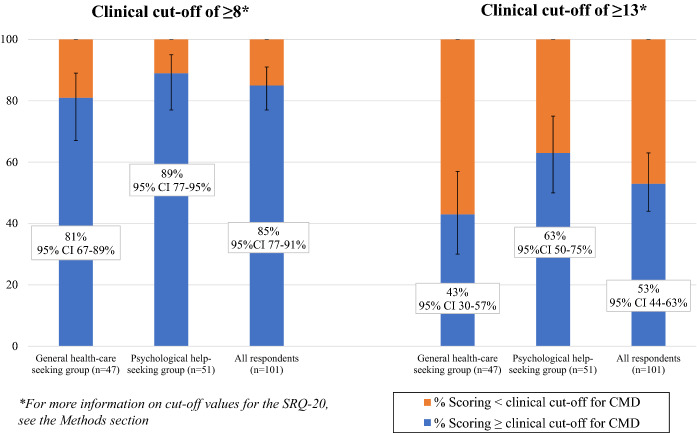
Percentage of Ums scoring above clinical cut-off on the SQR-20 for Common Mental Disorders (CMD). *SRQ-20* Self-Reporting Questionnaire, the 20 item version

In the general health care seeking group, 81% (95% CI 67–89%) scored above the SRQ-20’s cut-off value for CMD of ≥ 8, versus 89% (85% CI 77–95%) in the psychological help-seeking group. Of all participants together, this was 85% (95% CI 77–91%). If the more cautious value of ≥ 13 is used, 43% (95% CI 30–57%) of the general health care seeking group scored above the cut-off value, versus 63% (95% CI 50–75%) in the psychological help-seeking group (Fig. 2).

The mean SRQ-20 score was 11.6 ± 5.0 in the general health care seeking group and 13.1 ± 4.3 in the psychological help-seeking group (t(99) = − 1.73, *p* = 0.09). The mean SRQ-24 score was 12.4 ± 5.4 in the general health care seeking group and 14.6 ± 4.8 in the psychological help-seeking group (t(99) = − 2.17, *p* = 0.03), indicating that groups differed mostly on the presence of psychotic experiences (Table 2, see also Fig. 2).

**Table 2:** Mean scores SRQ-20 and SRQ-24

Questionnaire	General health care seeking group n = 47	Psychological help-seeking group n = 54	*P* value
SRQ-20	11.6 ± 5.0*	13.1 ± 4.3	0.087
SRQ-24	12.4 ± 5.4	14.6 ± 4.8	0.033

*SRQ-20* Self-reporting questionnaire, 20-item version. *SRQ-24* Self-reporting questionnaire, 24-item version

*Mean test scores are denoted as mean ± 1 SD

**Fig. 3:**
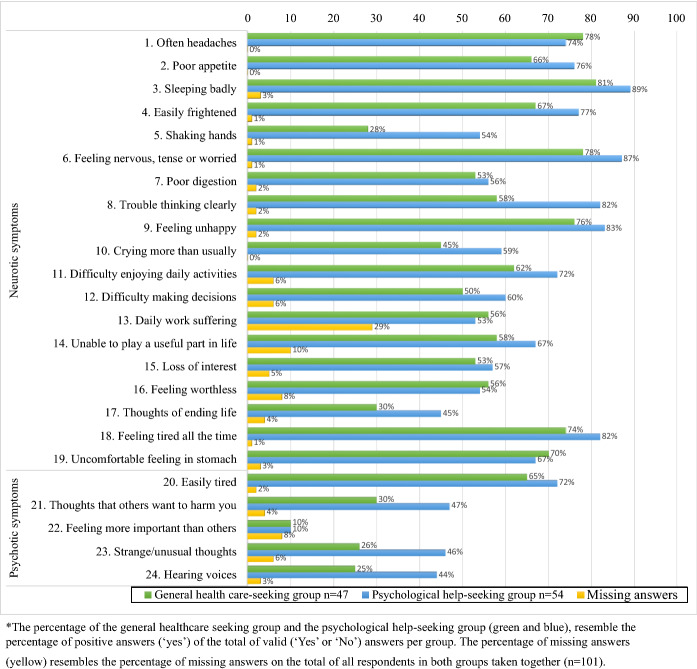
Percentage* of symptoms present on SRQ-24 per group

The two most reported symptoms were sleeping badly (84%) and feeling nervous, tense, or worried (82%). Thirty-seven percent of the respondents had thought about ending their life in the past thirty days. Thirty-five percent reported hearing voices that others could not hear (Fig. 3).

In 33% of the medical records of the participants, a previous psychiatric history was recorded, meaning a previously diagnosed psychiatric disorder, current or previous use of psychotropics, a previous psychiatric treatment or a suicide attempt.

### Problems in Help-Seeking UMs

In 36 of 99 (36%) retrievable medical records, adverse life events were documented by the physician or psychologist. In the psychological help-seeking group this was 33 out of 54 (61%), versus 3 out of 45 (7%) in the general health care seeking group. Some respondents reported more than 1 event. In total, 49 events were listed. The most reported events were physical assault (including torture), sexual assault, captivity, and combat or exposure to a war-zone (Table 3).

**Table 3:** Adverse life events

Category*	*n*	Example quote**
Physical assault	13	“Tortured by Al-Shabaab”
Sexual assault	10	“Raped multiple times, was in prison in Libya”.
Captivity	8	“Held captive in mortal danger”.
Combat or exposure to a war-zone	6	“He thinks about history, about the war in Somalia. Wakes up scared often. Had to flee, people around him were shot dead”.
Any other very stressful event or experience	5	“Experienced how a woman had to give birth on a plastic boat at open sea”.
Sudden violent death	4	“In the past, she saw her parents get killed”.
Sudden accidental death	2	“Mother died during travels to Netherlands”
Transportation accident	1	“Fled to Libya, crossed over by boat to Italy; the boat capsized causing twenty people to perish. Patient himself was rescued from the water after 20 h”.

*Events were categorized using the ‘Life Events Checklist for Diagnostic and Statistical Manual of Mental Disorders-5

**Quotes are translated texts from electronic patient records from visits from the general medical consultation hour and the Psychosocial Support Program

SRQ-24 scores were significantly higher for respondents who reported adverse life events (mean = 15.9 ± 4.6) than respondents who did not (mean = 12.2 ± 5.1); t(97) = − 3.61, p < 0.001. Effect size was 0.76, indicating a medium-to-large effect. No statistically significant associations were found between SRQ-24 scores and sex, age, length of stay in the Netherlands, presence of chronic disease, psychiatric history, substance use, living conditions, and region of origin.

### Mental Health Referrals

Twenty-nine percent of all respondents were referred to a mental health service outside of the non-governmental organization. In the general health care seeking group this was 28% versus 30% in the psychological help-seeking group. Referred respondents had significantly higher SRQ-24 scores (mean = 16.1 ± 5.5) than those who were not referred (mean = 12.6 ± 4.8); t(99) = − 3.21, *p* < 0.001. Effect size was 0.68, indicating a medium-to-large effect.

### Additional Results: Psychosocial Support Program

The Psychosocial Support Program that was initiated in June 2017 as a response to the preliminary results of this study is still in place. Annually, the number of consultations is increasing. In 2019, 416 consultations took place. In 2020, 1108 consultations were recorded. In the first quartile of 2021, there have been 327 consultations, indicating a further increase of the use of the service. No data are available on 2017–2018.

## Discussion

### Summary of Major Findings

This study examined the prevalence of mental health problems in UMs (*N* = 101) visiting a low-threshold primary health care service provided by an NGO. The prevalence of mental health problems and the number of referrals to mental health care services were high, also in respondents that primarily presented with physical problems only. A prevalent factor that significantly influenced mental health problems was a history of adverse life events, of which physical assault, sexual assault, captivity, and combat or exposure to war-zone were most common. Poor living conditions were common but not significantly related to mental health outcomes in this sample.

### Findings in Relation to Other Studies

The 81% (95% CI 67–89%) of UMs at the general medical consultation hours scoring above the cut-off value on the SRQ-20 is high. We hypothesize that the high prevalence of CMD in our study might be explained by the specific issues that come with being undocumented: social exclusion, bad housing conditions, lack of emotional support, uncertainty about legal status, fear of being arrested, unemployment and exploitation, poor health, and difficulty accessing health care. Often these issues are longlasting and lacking prospects of improvement (Chauvin and Simonnot 2013; Lahuis et al. 2019; Rietveld et al. 2019; Teunissen et al. 2014a, b; Woodward et al. 2014). This study also showed a high prevalence of potentially traumatic adverse life events, and a correlation between these adverse life events and SRQ scores.

Several studies describe poor self-reported mental health in UMs (Woodward et al. 2014). In one study, physicians and patients called the generalized stress and anxiety affecting health the ‘illegal syndrome’ (Castañeda 2009). A study performed in Switzerland showed that migrants who were undocumented had worse mental health than migrants who were in the process of regularization. However, the absence of a residency permit was in itself not a significant predictor, after adjusting for measures of integration, social support, and economic resources. However, social isolation, exposure to abuse or discrimination, and lack of financial resources were significantly associated with worse mental health (Fakhoury et al. 2021).

In a descriptive study among hundred female UMs in The Netherlands, more than 70% reported psychological problems including anxiety and sleeplessness (Schoevers et al. 2009). In a European survey study, 22.7% of the UMs in Amsterdam reported a poor to very poor mental health, compared to 4.9% of the general population (Chauvin and Simonnot 2013). On the other hand, the prevalence estimates of mental health problems in UMs vary considerably. Two retrospective studies in the Netherlands found that actually fewer mental health problems were registered in medical records from UMs compared to those from migrants with a residence permit (Teunissen, van den Bosch et al. 2014a, b; Wolfswinkel et al. 2009). Also, the levels of psychiatric disorders among UMs were not elevated compared to the general population (Teunissen, van den Bosch et al. 2014a, b; Wolfswinkel et al. 2009).

The high prevalence of mental health problems observed in this study may be explained by several factors. Firstly, the current sample differs from studies conducted in general practices. Most of the participants in this study were probably not registered with a general practice, as they were visiting the health care service of DotW. Not being registered in a general practice could be correlated to having a smaller support network, thus influencing mental health negatively. A study of Schoevers et al. among undocumented women found that most women found their GP with the help of a voluntary support organization (Schoevers et al. 2010). It is likely that being supported by such organisations positively affects their social network and mental health. Secondly, while UMs themselves do not tend to easily report existing mental health issues (Teunissen et al. 2014a, b), volunteering health care professionals at DotW may be more focussed on identifying these than regular health care professionals, because of the expertise of DotW with this population. In addition, general practitioners tend to underreport mental health problems of UMs in their medical files (Teunissen et al. 2015). Thirdly, it is likely that the group of UMs visiting DotW included a larger proportion of rejected asylum seekers compared with the total group of UMs in the Netherlands. An earlier report showed that 67% of the patients of DotW consists of rejected asylum seekers, which is considerably higher than the most recent (but dated) estimation of 11–33% of UMs in the Netherlands (Dokters van de Wereld 2018; van der Leun and Ilies 2008). It is plausible that rejected asylum seekers are more vulnerable to mental health problems than other UMs, like people overstaying work permits or visas. A meta-analysis in 2009 showed that the prevalence estimates of major depressive disorder and anxiety disorder were almost twice as high among refugees as among labour migrants (Lindert et al. 2009). On the other hand, the stigma associated with psychological difficulties might have led to an underestimation of the prevalence of mental health problems in our study due to under-reporting (Cha et al. 2019; Kirmayer et al. 2011; Priebe et al. 2016; Teunissen et al. 2014a, b).

We found a high number of adverse life events in our study. This is even more remarkable as respondents were not systematically asked about life events. In addition, consultations took place by rotating medical staff at a hardly convenient facility, which possibly contributed to under-reporting. However, retrospective reporting can also lead to over-reporting due to demoralization and due to active searching for explanations for the current state of mind. Despite this potential imprecision, this study found high numbers of life events which had a medium-to-large effect on symptoms of common mental health disorders. Several other studies emphasize the vulnerability of UMs and refugees to physical and sexual assault (Chauvin and Simonnot 2013; Keygnaert et al. 2015; Schoevers et al. 2009).

Counterintuitively and contrasting with the subjective experience of UMs themselves as found in other studies (Cha et al. 2019; Teunissen et al. 2014a, b), we found no significant correlation between poor living conditions and mental health. This may be related to the small sample size in our study.

### Strengths and Limitations

Firstly, a strength of this study is the unique access to this population of UMs. The sample comprised a diverse group of UMs with regard to living situation (including night shelters, squats, living on the streets) and country of origin. Secondly, the study used a dependable and widely used questionnaire to assess mental health, which screens for (subclinical) mental health problems instead of using higher threshold criteria which are often used for diagnostic framing. This more adequately captures the full range of severity of mental health problems. Thirdly, a thorough electronic patient record search was conducted, including an assessment of adverse life events.

However, certain limitations need to be addressed. This study has a limited statistical power due to a small sample size and therefore small subgroups, which limited our ability to explore the association of living situations and country of origin with mental health. The convenience sampling method of a help-seeking population limits generalizability. This is illustrated by the fact that regions of origin are distributed differently than in the most recent estimation report about UMs, indicating that there is underrepresentation of certain groups in this study. In this estimation report, most people originated from Europe, North Africa, and Asia, while in our study, the largest group originated from Sub-Saharan Africa (Heijden et al. 2020). In addition, the fact that the respondents were seeking for medical help may have led to a higher prevalence estimate of mental health problems. On the other hand it must be noted that when DotW’s volunteering staff visit a location with the van, they usually chat informally with the residents and actively invite them to attend the consultation hour if needed. This means that many UMs come with relatively ‘small’ complaints or questions, like having a runny nose since a few days, or asking for a tooth brush of hygienic pads. In other words, our study population was not really actively seeking help, which may have reduced the prevalence estimates. To a certain extent this kind of bias is inevitable, since due to the hidden nature of the group and the lack of available data about the population of UMs it is virtually impossible to conduct random sampling.

Several factors might have led to an imprecision of results. The SRQ is not validated for a comparable population, i.e. an ethnically diverse group of UMs in a Western country. Validation studies in different populations have led to broadly varying cut-off values, ranging from ≥ 3 in Ethiopia to ≥ 17 for women in Afghanistan (Hanlon et al. 2008; Ventevogel et al. 2007). We tried to address this limitation by using the most used cut-off value of ≥ 8 and a more cautious value of ≥ 13 (Rahman and Hafeez 2003). Some questions of the SRQ did not fit our population, reflected by high numbers of missing answers for two questions, namely: ‘Is your daily work suffering?’ (29% missing) and ‘Are you unable to play a useful part in life?’ (10% missing). This is probably related to the high unemployment rate in UMs (Chauvin and Simonnot 2013).

Last, due to the retrospective assessment of the electronic patient records, the number of adverse life events is probably an underestimation.

## Implications for Clinicians and Policymakers

This study illustrates the need for social and psychological support for UMs, and therefore underlines the urgency of improving access to mental health care.

### Clinician Level

We hope this study will increase awareness among GPs and other health care professionals of the prevalence of mental health issues among UMs. We recommend health care providers to actively explore such problems, as UMs tend to not bring these up themselves (Teunissen et al. 2014a, b). In addition, it is important that clinicians are able to work culturally sensitive to ensure appropriate care. Health care providers, but also front office workers of general practices and hospitals, should have sufficient knowledge of the entitlements of undocumented people. Lack of knowledge of professionals should not be a barrier for UMs to receive health care. Online trainings exist to train professionals on these issues (Johannes Wier Stichting 2018).

### Health System Level

With regard to access to mental health care, support programs provided by NGOs appear to be an important first step, but more structural and institutionalized efforts are required. Striving for the registration of all UMs with a GP is key to guarantee access to primary health care as well as timely specialist care when needed. In addition, given the impact of language barriers on the accessibility, acceptability, and quality of care, the government should ensure free and easy access to professional interpreter services for all professionals in medical and social care. A positive recent change is that costs for interpreters in mental health care for adults are covered by health insurance since the beginning of 2022. However, for other sectors of health care this is still not the case.

### Policy Level

In order to improve health outcomes, a more integrative approach is required by addressing the societal structures that may provoke or nourish mental health problems among undocumented migrants. The Dutch governmental attitude towards UMs is characterized by exclusion and discouragement of ‘unlawful residence’, which is organized through three channels (van der Leun and Ilies 2008). Firstly, access to the formal labour market is blocked, as employers risk high fines by employing UMs. Secondly, “The Linkage act” excludes undocumented people from a spectrum of social services, including social benefits and a health insurance (van der Leun and Ilies 2008). Thirdly, undocumented migrants face the risk of detention and expulsion (van der Leun and Ilies 2008). In these detention centres, UMs experience being treated similarly to criminals, which poses a risk for retraumatization (Boersma et al. 2022).

Economic considerations seem to play a minor role in these policies. In a recent study, respondents from the Dutch ministry of Health Welfare and Sport stated it would probably be cheaper for the Dutch government if UMs could insure themselves instead of relying on a reimbursement fund, and international research supports the claim that restricting access to health care results in higher costs in the long term (Bozorgmehr and Razum 2015). However, in the political debate, discouraging immigration seems to be considered as more important than economic or health considerations (Grit et al. 2012).

Instead of building policies around discouragement, the authors plea for human rights as a guiding principle in arranging entitlements and services for UMs, particularly the right to health, the right to work, and the right to living in dignity. In line with this, being able to acquire health insurance should not depend on legal status.

Being able to participate in society by working is vital for health and protects against exploitation. An intermediary step regarding the right to work could be to enable participation, e.g. through partaking in volunteering jobs, following courses or educational programs, as advocated by human rights groups (Iedereen aan de slag! 2019). Some positive changes on local level have already taken place, such as the extension of educational opportunities for UMs in several larger Dutch municipalities (Hielkema 2022).

## Suggestions for Future Research

In general, we advise to include input of UMs themselves in study design and research activities when studying UMs. Practical advice on performing research ethically with undocumented migrants is given in the article of Van Den Muijsenbergh (Van Den Muijsenbergh et al. 2016). To get a more precise idea about prevalence of mental health issues among the group of UMs and potential determinants of mental health, we recommend a larger, and ideally longitudinal study with follow-up measurements targeting a non-health care seeking population, for example, UMs visiting a community centre. However, given the nature of this group, it is questionable whether performing longitudinal research is achievable. In addition, we suggest to not just assess factors that might influence mental health negatively, but also examine positive and protective health factors (factors that could contribute to well-being and protect against mental health problems), e.g. adequate housing, social networks, psychosocial support, and obtaining a legal status.

Moreover, future studies might explore the experience of UMs and barriers to access ‘alternative care’ (‘safety net care’) as usually provided by NGOs such as—but not limited to—DotW.

Lastly, as already much is known about the existing barriers to (mental) health care, future studies might focus on the implementation of widely proposed policy recommendations to improve access to health care for UMs.

## Conclusion

This study is one of the first to examine the burden of mental health problems among help-seeking UMs (N = 101) visiting a low-threshold health care service provided by a NGO. The prevalence of mental health problems and the number of referrals to mental health care services were high, also in respondents that primarily came to seek health care for physical problems. Of all participants, 85% (95% CI 77–91%) scored at least or above the clinical cut-off value for common mental disorders on the SRQ-20. These findings imply that mental health problems are widespread among help-seeking UMs. A Psychosocial Support Program was initiated based on preliminary results of this study, and seems to meet a need, since it is visited frequently and the amount of patients is increasing annually. A history of adverse life events significantly influenced mental health problems in UMs in this study, of which physical assault, sexual assault, captivity, and combat or exposure to war-zone were the most prevalent. Poor living conditions were common, but in this sample not significantly related to mental health outcomes. Mental health problems of UMs are inextricably linked to concerns about legal status, unemployment and exploitation, deplorable housing conditions, social exclusion, and fear of detention and deportation. It is only by ameliorating these conditions that health outcomes can truly be improved. This requires above all political will to improve the situation of UMs, and thorough action from local and national governmental parties and NGOs.
